# Preparation of an Fe_80_P_14_B_6_ Bulk Nanocrystalline Alloy via Solidification from a Molten Alloy at Deep Undercooling

**DOI:** 10.3390/ma18061361

**Published:** 2025-03-19

**Authors:** Xiaoming Chen, Tuo Wang, Zhe Zhang, Yuluo Li, Mingming Wang, Kuang Lv, Guigen Wu, Xiaoli Wang, Zhangyin Li, Xidong Hui

**Affiliations:** 1 State Key Laboratory for Advanced Metals and Materials, University of Science and Technology Beijing, Beijing 100083, China; 2 Key Laboratory of Surface Engineering of Equipment for Hydraulic Engineering of Zhejiang Province, Standard and Quality Control Research Institute, Ministry of Water Resources, Hangzhou 310024, China; 3 Water Machinery and Remanufacturing Technology Engineering Laboratory of Zhejiang Province, Hangzhou River Mechanical and Electrical Equipment Engineering Co., Ltd., Hangzhou 310024, China; 4 School of Materials Science and Engineering, Xinjiang University, Urumqi 830017, China; 5 Engineering Technology Research Institute, Chery Automobile Co., Ltd., Wuhu 241006, China

**Keywords:** Fe_80_P_14_B_6_ alloy, fluxing technique, deep undercooling, spinodal decomposition, nanocrystalline

## Abstract

Using fluxing technology, molten Fe_80_P_14_B_6_ alloy achieved significant undercooling (Δ*T*). Experimental results demonstrate that the solidified morphologies of the Fe_80_P_14_B_6_ alloy vary considerably with Δ*T*. At Δ*T* = 100 K, the microstructure is dendritic. At Δ*T* = 250 K, a variety of eutectic morphologies are observed, including a network-like structure near the solidification center, attributed to liquid spinodal decomposition. At Δ*T* = 350 K, the microstructure exhibits a uniform, random network-like morphology with approximately 50 nm. The mechanical property of the specimens solidified at different Δ*T* was checked by microhardness test, indicating that the hardness of the specimens increases with the increase in ΔT, reaching a maximum value of 1151 HV_0.2_.

## 1. Introduction

Nanostructured alloys, first proposed by Gleiter [1] and Turnbull [2], have attracted significant attention due to their promising properties [3,4]. To date, bulk nanostructured alloys can be produced using various techniques, which can be broadly categorized into two main approaches. The first approach, introduced by Gleiter and colleagues [5] involves the preparation of nanometer-sized powders via evaporation techniques, followed by high-pressure sintering to form bulk specimens. The second approach involves the preparation of bulk nanostructured alloys using annealing bulk metallic glasses at an elevated temperature [6]. In principle, bulk nanostructured alloy can also be prepared by the direct solidification of a molten alloy subjected to deep undercooling [4,7,8], and via such a route, the liquid phase spinodal decomposition (LSD) may occur in some appropriate systems [9,10]. Another way of preparing compact nanophase alloys by melt-quenching in a single step was described by Bakonyi and Cziraki [11].

Kui and Guo [7] observed the LSD phenomenon during the solidification process of an undercooled eutectic Pd_82_Si_18_ molten alloy. A similar phenomenon was also found in the eutectic alloy Pd_40.5_Ni_40.5_P_19_ [4,12,13]. However, the heats of mixing between the main elements such as Pd-Si, Pd-P and Ni-P are all negative in these two eutectic alloys [14]. Typically, the LSD phenomena occur only in systems with a positive heat of mixing among the main elements. Due to a lack of direct evidence and supporting theory, liquid phase separation in alloys with a negative heat of mixing has become a controversial issue. Kui [15,16,17,18] proposed that there is a miscibility gap in undercooled molten eutectic alloys. When a homogeneous eutectic alloy melt is undercooled into its miscibility gap, it decomposes into multiple liquid sub-networks of a characteristic wavelength *λ* that depends critically on the level of undercooling at which the decomposition takes place. When the undercooling is sufficiently large, *λ* can be smaller than 100 nm. Surface tension then sets in, breaking up the networks into droplet shapes. If crystallization is bypassed, it becomes a bulk amorphous nanostructure. If crystallization cannot be avoided, it will take place inside the miscibility gap, to transform the system into a nanostructure with a crystalline solid of network-like morphology. Guo et al. [18] have demonstrated fully such phase evolution in the undercooled Pd_82_Si_18_ eutectic alloy. As a result, a nanostructured Pd_82_Si_18_ alloy with grain size 3~6 nm was prepared. Therefore, the LSD can afford an effective method to produce nanostructured materials with tailorable grain size.

The LSD phenomena have been reported in some iron-based systems. Ho et al. [15,16] were able to cast molten Fe-C alloys into ingots of network-like morphology, which have attractive mechanical properties. For example, Fe_83_C_17_ network alloys have a compressive yield strength of ~2000 MPa, a compressive strength of ~2500 MPa, a plastic strain to failure of ~18 pct, and a hardness value of ~536 HV. Chow et al. [17,19] found that an undercooled Fe_79.5_B_6.5_C_14_ molten ingot can also be cast into a network ingot and its compressive strength can be as large as ~3700 MPa, a hardness value of ~800 HV.

Both the Pd_82_Si_18_ and Fe_80_P_14_B_6_ alloy systems are all the “metal-metalloid” type. In addition, Fe_80_P_14_B_6_ alloy can be regarded as a pseudo-binary eutectic alloy with the composition of Fe_80_(P,B)_20_, of which the equilibrium phase diagram is similar to that of Fe-P binary eutectic alloy. Thus, it can be expected that Fe_80_P_14_B_6_ alloy may also undergo the LSD when the undercooling is deep enough. In this work, in order to obtain bulk nanostructured metals with better performance, based on Fe_80_P_14_B_6_ alloy, we attempt to prepare bulk nanocrystalline alloys with controllable grain size and uniform distribution of grain size by metastable LSD mechanism.

## 2. Experiments

Fe_80_P_14_B_6_ alloy ingots were prepared from iron powder (99.98% pure), Fe_3_P powder (98% pure), and boron powder (99.95% pure) (Alfa Aesar, Shanghai, China). After the right proportion was weighed, they were put into a clean fused silica tube and alloyed by torch under an argon atmosphere. Then, the as-prepared alloy ingots were fluxed in molten B_2_O_3_ about 4 h under a vacuum of ~10 Pa. After the fluxing treatment, the whole system was transferred to the furnace and placed on a thermocouple. The thermocouple and computer were used to record the temperature of the molten specimen. As soon as a crystallization event occurred, the whole system was removed from the furnace and quenched into the iced water. The diameter of each sample is about 2.5~3.0 mm. There was a shrinking point that could be regarded as the solidification center when the sample had solidified. The solidification center was marked by a color pen, and then the sample was cut from the center along the diameter by a wire cutting machine. The phase structure of the samples was characterized by X-ray diffractometer (XRD) using a Bruker D8 ADVANCE (Cu Kα radiation) (XRD, Bruker, Berlin, Germany). The microstructures and the phase composition of the specimens were investigated using a SUPRA55 FE-SEM (ZEISS, Oberkochen, Germany) with energy dispersive X-ray spectrometers (EDS, X-MaxN20, Oxford, UK). For SEM analysis, the samples were etched with a solution of CH_3_CH_2_OH and HNO_3_ in a volume ratio of 9:1. Vickers microhardness was measured under 200 g load with 10 s dwell time (HXD-1000TMC, Taiming, China, 3 samples for each case, five indents per sample).

## 3. Results and Discussion

### 3.1. The Evolution of the Solidified Morphology Under Different Levels of Undercooling

The fluxing technique was employed to undercool the molten alloy Fe_80_P_14_B_6_ below its melting temperature in the present work. The melting temperature (*T_m_*) of Fe_80_P_14_B_6_ alloy was determined to be 1310 K by Differential Thermal Analysis (DTA). If *T* is referred to as the crystallization temperature of the specimens, then the crystallization undercooling is defined as Δ*T* = *T_m_*–*T*.

**Figure 1:**
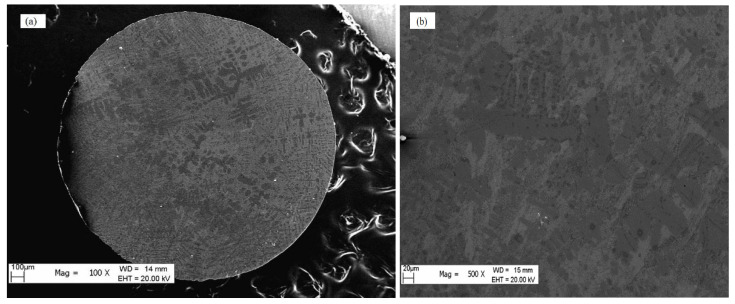
(**a**) The morphology of Fe_80_P_14_B_6_ alloy sample solidified at the undercooling of Δ*T* = 100 K under low magnification SEM. (**b**) The primary dendrites under high magnification.

When Δ*T* = 100 K, Figure 1a shows the general morphology, and a primary dendrite morphology is observed under low magnification. Figure 1b shows the primary dendrites and the matrix under high magnification. The dendrites are composed of α-Fe, and the matrix is composed of α-Fe and Fe_3_(P,B), as determined by EDX.

**Figure 2:**
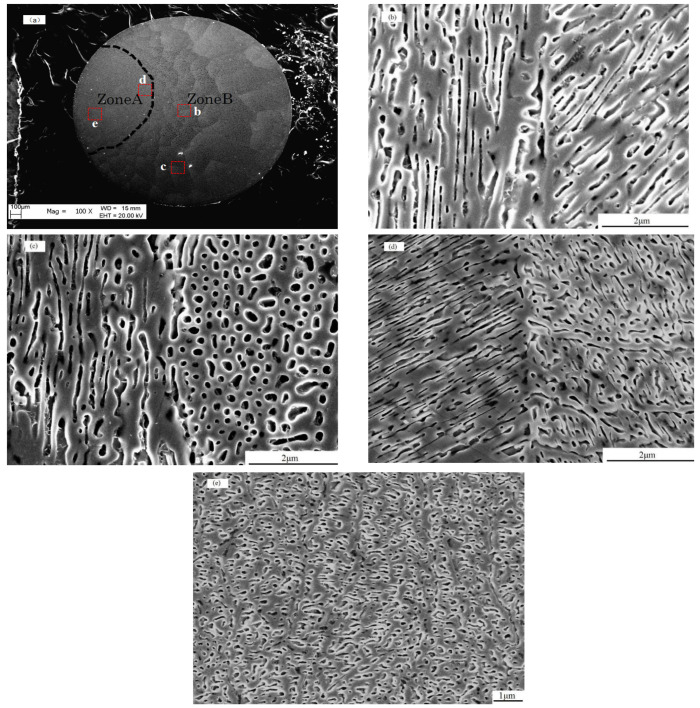
(**a**) The morphology of Fe_80_P_14_B_6_ alloy sample solidified at the undercooling of Δ*T* = 250 under a low magnification SEM observation shows zones A and B. (**b**) One type of patch boundary of Zone B at the undercooling of Δ*T* = 250 examined under an FE-SEM. (**c**) Another type of patch boundary of Zone B at the undercooling of Δ*T* = 250 K examined under an FE-SEM. (**d**) The morphology of Zone A at the undercooling of Δ*T* = 250 K examined under an FE-SEM. (**e**) The random two-phase network morphology in Zone A at the undercooling of Δ*T* = 250 K examined under an FE-SEM.

When Δ*T* = 250 K, a low magnification SEM image is shown in Figure 2a. There are two zones (dash line) in the image. While Zone A is very smooth, there are some cell-like features in Zone B. In fact, there are quite different morphologies in these zones. Figure 2b–e is an FE-SEM micrograph under a high magnification, showing the patch boundaries and morphologies of each side. There are two types for the patch boundaries in Zone A. One is Figure 2b. It shows a typical morphology about lamellar eutectic, and each side of the boundary shows a strong and different direction for the lamellar. The dark lamella is composed of α-Fe and that of the bright area is composed of Fe_3_(P,B) which can be checked by EDX. The composition of the precipitated phase is Fe92(PB)8 and the matrix is Fe72(PB)28. Figure 2c shows another patch boundary and morphologies for both sides. The morphology shows a boundary between the lamellar eutectic and rod eutectic. The bar is composed of α-Fe, and the bright area is composed of Fe_3_(P,B). Zone B (Figure 2d) is another boundary. We can see that the morphology on the left is the lamellar with a strong direction, but on the right, it becomes a random network-like microstructure whose morphology is like liquid spinodal decomposition, as suggested by Cahn [20,21,22,23]. The network (Figure 2e) is composed of two sub-networks, and the wavelength of one network is about 200 nm and that of another one is about 250 nm. The smooth area is composed of Fe_3_(P,B), and the dark area is composed of α-Fe.

**Figure 3:**
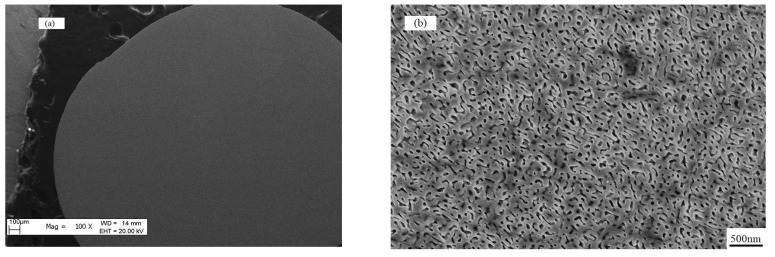
(**a**) The morphology of Fe_80_P_14_B_6_ alloy sample solidified at the undercooling of Δ*T* = 350 K with a low magnification SEM observation. (**b**) The uniform and random network structure formed by spinodal decomposition at the undercooling of Δ*T* = 350 K under an FE-SEM.

When Δ*T* = 350 K, from the low-magnification SEM image (Figure 3a), one can see that the microstructure is very uniform. The micrograph under a high magnification FE-SEM (Figure 3b) shows that there are no eutectic morphologies, but rather a structure of interconnected networks on the whole surface of the sample. The morphology of the networks is very uniform and random. The wavelength of the network is about 50 nm. The result of EDX shows that the dark network and bright network are composed of α-Fe and Fe_3_(P,B) phases, respectively.

**Figure 4:**
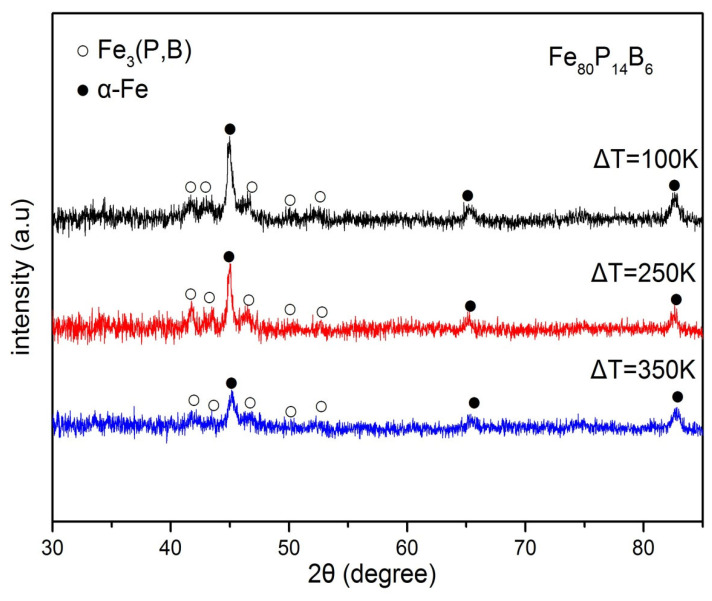
XRD patterns of Fe_80_P_14_B_6_ alloy samples solidified at the different undercooling Δ*T.*

At the same time, the XRD study (Figure 4) also suggests that the phases of the samples at ΔT = 100 K, 250 K, 350 K all consist of α-Fe and Fe_3_(P,B) phases, which is consistent with the above results of EDX analysis.

The ternary Fe_80_P_14_B_6_ alloy can be considered as a pseudo-binary eutectic alloy with the composition of Fe_80_(P,B)_20_. For a eutectic system, if the temperature is high, the entropy is the dominant for the Gibbs free energy, resulting in a homogeneous liquid phase. However, when the temperature is low enough, the enthalpy will become the dominant, so there will be a miscibility gap for the liquid phase at the larger enough undercooling.

**Figure 5:**
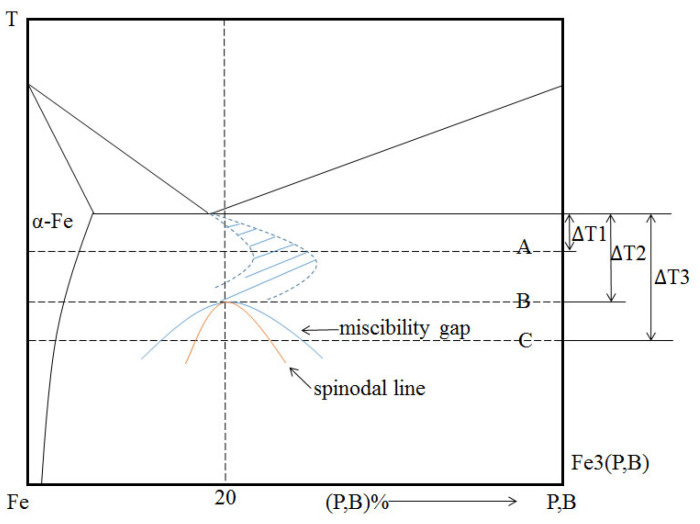
Schematic diagram about the phase diagram of pseudo-binary eutectic Fe-(PB) alloy to illustrate the present experimental results.

From the XRD (Figure 4), we can see that the Fe_80_P_14_B_6_ alloy specimens solidified at the different undercoolings all consisted of α-Fe and Fe_3_(P,B) phases. So, a schematic phase diagram is shown in Figure 5 for the present Fe_80_P_14_B_6_ alloy based on the above discussion. With the condition of non-equilibrium solidification, the eutectic area of this metal-metalloid alloy under the undercooling presents an asymmetric coupled zone, as shown in the dash zone in Figure 5.

When Δ*T*_1_ = 100 K (sample A), the undercooling is far away from the miscibility gap. The skewed couple zone dictates that only the α-Fe phase can grow as a primary phase in this region for this composition, so α-Fe becomes the primary dendrite. When Δ*T*_2_ = 250 K (sample B), the molten sample just reach the miscibility gap so we can see the random network-like in Zone A. However, the released latent heat of solidification in Zone A will transfer to Zone B, resulting in the increase in temperature in Zone B, in which the temperature may move out of the miscibility gap to the pseudo-eutectic area. As a result, the undercooling in Zone B will decrease. In addition, the heat conduction can make a temperature gradient in Zone B, which will lead to the nuclear driving force decreasing in different degrees, and the proportion of α-Fe and Fe_3_(P,B) phases may also change. So, a variety of eutectic morphology can be found in Zone B. When Δ*T*_3_ = 350 K (sample C), the undercooling is large enough. Therefore, the released latent heat at the solidification center is not enough to raise the temperature outside the spinodal line. Thus, Sample C presents a uniform random network-like microstructure for all regions as shown in Figure 3b.

### 3.2. The Evolution of Microhardness Under Different Levels of Undercooling

**Figure 6:**
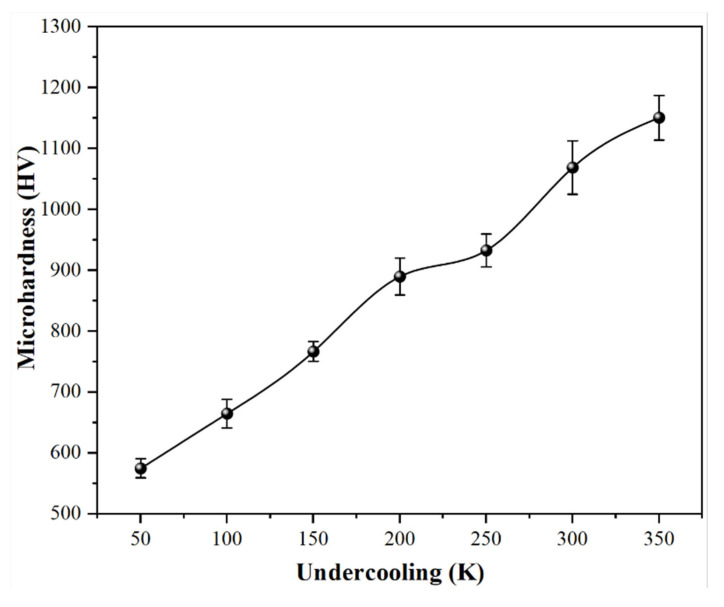
The microhardness of Fe_80_P_14_B_6_ alloys solidified at the different undercooling.

The microhardness of each sample solidified at the different undercooling was determined by a micro hardness tester. The results (Figure 6) show that the microhardness of the samples increases with the undercooling. The microhardness rises to as high as 1151 HV_0.2_.

It has been considered that the grain size of the samples decreases with the undercooling. The smaller the grain size, the more the grain boundaries, which leads to greater resistance for the deformation of the material. In addition, it is more important that the samples solidified under deep enough undercooling will present a network-like microstructure, which may result in a high hardness. Based on the above reasons, it can explain why the hardness increases with the undercooling increases.

## 4. Conclusions

With the help of fluxing technique, the molten Fe_80_P_14_B_6_ alloys can be solidified at different undercooling (Δ*T*) through isothermal undercooling experiments. The crystalline morphologies of the Fe_80_P_14_B_6_ alloys solidified at different undercooling are quite different. When Δ*T =* 100 K, the traditional crystalline morphology i.e., α-Fe primary dendrite can be found. When Δ*T* = 250 K, a random network-like region and patch boundaries with different eutectic microstructures can be found. When Δ*T* = 350 K, the solidification morphology is uniform random network for all regions, which is controlled by LSD mechanism, and the average grain size is about 50 nm. The mechanical property indicated that the microhardness of the Fe_80_P_14_B_6_ alloy increases with the increase in the undercooling.

## Data Availability

The original contributions presented in this study are included in the article. Further inquiries can be directed to the corresponding authors.
